# Co-Amorphization of Acemetacin with Basic Amino Acids as Co-Formers for Solubility Improvement and Gastric Ulcer Mitigation

**DOI:** 10.3390/pharmaceutics16060745

**Published:** 2024-05-31

**Authors:** Jiayue Hou, Peixu Zhao, Yanfei Wang, Xiwei Jiang, Qiang Fu

**Affiliations:** 1 Wuya College of Innovation, Shenyang Pharmaceutical University, No. 103, Wenhua Road, Shenyang 110016, China; 2 College of Medical Equipment, Shenyang Pharmaceutical University, No. 103, Wenhua Road, Shenyang 110016, China; 3 Joint International Research Laboratory of Intelligent Drug Delivery Systems, Ministry of Education, Shenyang 110016, China

**Keywords:** acemetacin, co-amorphous, basic amino acids, gastric ulcer

## Abstract

Acemetacin (ACM) is a new non-steroidal anti-inflammatory drug with anti-inflammatory, analgesic, and antipyretic effects. However, the poor water solubility and gastrointestinal side effects limit its use. Recently, the co-amorphous (CAM) strategy has attracted great interest to improve solubility for poorly water-soluble drugs, and basic amino acids have the potential to protect the gastrointestinal tract. In order to develop a highly efficient and low-toxic ACM formulation, we prepared ACM CAM systems, with basic amino acids (lysine, arginine, and histidine) as co-formers, using a cryo-milling method. The solid-state behaviors of the ACM CAM systems were characterized by polarizing light microscopy, differential scanning calorimetry, and powder X-ray diffraction. Fourier transform infrared spectroscopy and molecular docking were carried out to understand the formation mechanism. Moreover, the gastro-protective effects of ACM CAM systems were evaluated in a rat gastric ulcer model. The results demonstrated that the CAM systems improved the dissolution rates of ACM compared with the neat amorphous counterpart. Furthermore, ACM CAM systems are significantly effective in mitigating the ACM-induced gastric ulcer in rats, and the ulcer inhibition rates were almost 90%. More importantly, this study provided a useful method for mitigating drug-induced gastrointestinal damage and broadened the applications of drug–amino acid CAM systems.

## 1. Introduction

**Figure 1:**
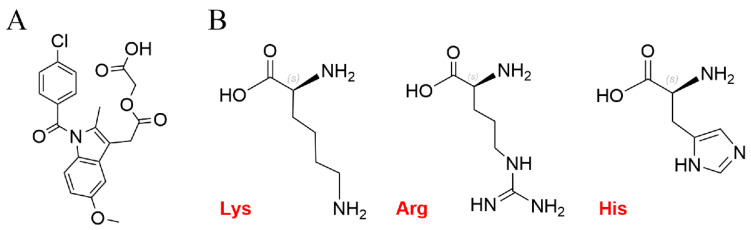
Molecular structures of (**A**) ACM and (**B**) Lys, Arg, and His.

Acemetacin (ACM) (Figure 1A) is a new non-steroidal anti-inflammatory drug (NSAID) with anti-inflammatory, analgesic, and antipyretic effects. It is a biopharmaceutics classification system class II drug, and its poor water solubility hinders oral bioavailability [1]. However, ACM may cause gastrointestinal damage [2] despite the increased gastrointestinal tolerance [3], because it is a prodrug of indomethacin [4]. Therefore, how to improve the solubility and eliminate the negative effects of ACM has become a key issue for clinical application.

The co-amorphous (CAM) system is a promising approach to improve the solubility and bioavailability of poorly water-soluble drugs. The CAM system is a homogeneous amorphous single-phase system composed of two or more low-molecular weight components [5]. Co-formers (citric acid, lactose, mannitol, amino acids, alginate, etc.) are usually used to stabilize the CAM system by the formation of hydrogen bonds or charges with the drug. Among these co-formers, amino acids are the most widely used [6]. Especially basic amino acids such as lysine (Lys) [7,8], arginine (Arg) [8,9,10,11], and histidine (His) [8,12] (Figure 1B) have successfully been used as co-formers for acidic drugs. Recently, relevant reports showed that these basic amino acids have protective effects on the gastrointestinal tract [13,14,15,16,17,18]. For example, Lys forms aldehyde lysine catalyzed by lysyl oxidase and participates in the formation of connective tissues and also contributes to the protection of the gastric mucosa barriers [14,15]. Arg can generate nitric oxide under the synthase for vasodilation; thus, the integrity of the gastric mucosal barrier was maintained, and mucosal damage caused by gastric ulcers was reduced [16,17]. His can inhibit peptic ulcers induced by phytanergic nervousness, and it has a preventive and mitigating effect on the damage of the intestinal mucosa, because it is an effective scavenger of free radicals [13,18]. Therefore, these basic amino acids are promising co-formers for CAM systems to mitigate the gastrointestinal side effects of ACM.

In this study, to improve the solubility and mitigate the gastrointestinal side effects of ACM, ACM–basic amino acid co-amorphous formulations (ACM CAM systems) were formulated by a cryo-milling method. The physicochemical properties of ACM CAM systems as well as their in vitro dissolution were evaluated. Moreover, gastro-protective effect and histology studies of ACM CAM systems were also performed in rat gastric ulcer models to test the mitigation of gastrointestinal side effects. This study improved the compliance of ACM in clinical use and provided a promising method for mitigating drug-induced gastrointestinal damage.

## 2. Materials and Methods

### 2.1. Materials

ACM was purchased from Hubei Huizepu Pharmaceutical Technology Co., Ltd. (Wuhan, China). Lys, Arg, and His were purchased from Tianjin Damao Chemical Reagent Factory Co., Ltd. (Tianjin, China).

### 2.2. Preparation of ACM CAM Systems

The preparation of ACM CAM systems was carried out by a cryo-milling method using a GS60201 procedural cryogenic tissue grinder (Monad Biotech Co. Ltd., Suzhou, China). According to previous studies, when the molar ratio of ACM to basic amino acids is close to 1:1, the CAM system has the best thermal stability and the highest solubility [19,20]. Thus, ACM and the basic amino acids were weighed at a molar ratio of 1:1 and mixed for use. The mixtures were added into cryopreserved tubes (2.5 mL) with a 7 mm diameter steel ball. The samples were cooled with liquid nitrogen for 10 min. The cryo-milling was then carried out at a speed of 1300 rpm for 30 min with a 5 s pause at an interval of 30 s. The freezing and grinding process was repeated 3 times, and then ACM CAM system (ACM-Lys, ACM-Arg, ACM-His) samples were collected.

Amorphous ACM (ACM-AM) was prepared using the melt-quenching method. Appropriate amounts of ACM were weighed and melted completely at 180 °C. Liquid nitrogen was used for rapid cooling. After that, the sample was taken out and ground to obtain ACM-AM. To prepare physical mixtures (PMs) (PM-Lys, PM-Arg, PM-His), each basic amino acid (Lys, Arg, and His) and ACM were weighed and mixed in a 1:1 molar ratio. The samples were sealed in a foil bag and stored at room temperature. The ACM CAM systems can remain physically and chemically stable for up to 12 months.

### 2.3. Polarizing Microscope

The samples were characterized using an MP41 PLM instrument (Guangzhou MM Photoelectric Technology Co., Ltd., Guangzhou, China) controlled by the TCapture software (version 5.1.1). An appropriate amount of each sample was dispersed on a clean slide evenly. The samples were observed with a 50× scope under a dark field.

### 2.4. Powder X-ray Diffraction

Powder X-ray diffraction (PXRD) patterns were obtained using a D/MAX-2600 X-ray diffractometer (Rigaku Corporation, Tokyo, Japan) with Cu Kα radiation (λ = 1.5406 nm). The tube voltage was 40 kV, and the current intensity was 40 mA. The scanning range of 2θ was 5–40°, the scanning speed was 5°/min, and the step size was 0.05°.

### 2.5. Differential Scanning Calorimetry

Differential scanning calorimetry (DSC) was carried out using a DSC 250 apparatus (TA Instrument, New Castle, DE, USA) under a 50 mL·min^−1^ N_2_ gas flow. Approximately 4 mg of each sample was weighed and added into aluminum Tzero pans. The samples were heated for two heating cycles. First, the samples were heated to 180 °C at a ramp rate of 10 °C/min and then cooled down to 20 °C at a rate of 40 °C/min. After that, the samples were heated to 180 °C again at a ramp rate of 10 °C/min to determine the glass transition temperature (*T_g_*).

Moreover, *T_g (mix)_* was calculated by the Gordon–Taylor equation [21]. It was defined as the theoretical *T_g_* value of the amorphous system without any intermolecular forces. The equation is described as follows:

$$T_{g\;(\mathrm{mix})}=\frac{w_{1}\cdot Tg_{1}+k\cdot w_{2}\cdot Tg_{2}}{w_{1}+k\cdot w_{2}}$$

*T_g_*_1_ and *T_g_*_2_ are the values of the glass transition temperatures of ACM and amino acid, *w*_1_ and *w*_2_ are the weight fraction of components, and *k* is a constant. The constant k can be calculated by the following formula:

$$k=\frac{Tg_{1}\cdot \rho_{1}}{Tg_{2}\cdot \rho_{2}}$$

*ρ_1_* and *ρ_2_* are the densities of each component.

### 2.6. Investigation of Molecular Interaction

#### 2.6.1. Fourier Transform Infrared Spectroscopy

Fourier transform infrared spectroscopy (FTIR) spectra were obtained using a Nicolet iS20 FTIR (Thermo Fisher Scientific Co., Ltd., Waltham, MA, USA) equipped with an attenuated total reflectance accessory. Spectra were collected with OMMIC Software over the range of 4000–450 cm^−1^ (16 scans, resolution of 4 cm^−1^).

#### 2.6.2. Molecular Docking

The molecular docking of ACM and the basic amino acids was carried out using the AutoDock4.2.6 software package. The molecular structures of ACM, Lys, Arg, and His were downloaded from the Pub Chem database. Then, the global docking of ACM and three basic amino acids was performed using Autodock4.2.6 software. The grid spacing was maintained at 0.3472 Å, and the grid box was placed enclosing the binding pocket of ACM. The number of conformational searches was set to 100, and the lowest energy conformation was optimized.

### 2.7. Dissolution Tests

Dissolution studies were carried out using ZRS-8G dissolution equipment (Tianjin Tianda Tianfa Technology Co., Ltd., Tianjin, China). Dissolution tests were carried out using the paddle method. Each sample was added to the dissolution medium at a dosage of 30 mg. The dissolution tests were carried out in 900 mL of purified water, which was kept at 37 °C. The paddle was set at a rotation speed of 50 rpm/min. Ten milliliters of medium was removed from the dissolution cup at 5, 10, 15, 20, 30, 45, 60, and 90 min and replaced with an equal volume of purified water immediately. The medium was filtered through a membrane filter (syringe filter, 0.45 μm sterile) and diluted with an equal amount of anhydrous ethanol. The absorbance of the test solutions was measured at 319 nm using an I-6 ultraviolet–visible spectrophotometer (Nanjing Philes Instrument Co., Ltd., Nanjing, China). All the dissolution tests were performed in triplicate.

### 2.8. Intrinsic Dissolution Rates

The intrinsic dissolution rate (IDR) tests were conducted using an RC301 dissolution apparatus (Tianfa Analysis Instrument Co., Ltd., Tianjin, China). The samples (equivalent to 100 mg of ACM) were weighed and pressed into tablets using a micro-press at 50 bar for 1 min. The test was carried out in 500 mL of purified water at 37 °C. The rotation speed was set at 100 rpm/min. Ten milliliters of dissolution medium was sampled at 5, 10, 20, 30, and 60 min and replaced with the same volume of fresh water. The medium was filtered through a membrane (syringe filter, 0.45 μm sterile) and diluted with an equal volume of ethanol as the test solution. The measurement was carried out as described in Section 2.5. Each sample was measured in triplicate. The formula for calculating the IDR is as follows:

$$IDR=\frac{V\;dc}{dt}\times \frac{1}{A}$$

in which *V* is the volume of the dissolution medium, *A* is the area of the sample in contact with the dissolution medium (0.5 cm^2^), and *t* is dissolution time.

### 2.9. Gastro-Protective Effects

Animal studies were approved by the Animal Ethics Committee of Shenyang Pharmaceutical University (Shenyang, China) and carried out according to the Guidelines for the Care and Use of Laboratory Animals. Thirty male Sprague-Dawley (SD) rats (weighing 200 ± 10 g) were used in this study. The animals had free access to food and water.

The rats were randomly divided into eight groups and treated as follows: a blank control group received PBS (*n* = 3), a model group received ACM (dissolved in 20% (*m/v*) Tween 80) at a dose of 10 mg/kg (*n* = 4), PM-Lys group (10 mg/kg, *n* = 4), PM-Arg group (10 mg/kg, *n* = 4), PM-His group (10 mg/kg, *n* = 4), ACM-Lys group (10 mg/kg, *n* = 4), ACM-Arg group (10 mg/kg, *n* = 4), and ACM-His group (10 mg/kg, *n* = 4).

The animals received each treatment by oral gavage. Four hours later, the animals were euthanized, and the stomachs were carefully removed and opened along the greater curvature. PBS (pH 7.4) was used to wash the stomach. After that, the lesions were measured by photographing the area of the gastric ulcer using the ImageJ software (Java 1.8.0_345) (National Institutes of Health, Bethesda, MD, USA). The ulcer inhibition rate (UIR) was calculated as follows:

$$\mathrm{UIR}\;(\%)=\frac{A_{m}-A_{e}}{A_{m}}\times 100\%$$

*A_m_* is the average ulcer area of the model group; *A_e_* is the average ulcer area of the experimental group.

In addition, the gastric tissue samples of the 8 groups were immersed in 4% (*v*/*v*) polyformaldehyde for 24 h and then embedded in paraffin and cut into 4 μm thick slices. The slices were then stained with hematoxylin–eosin (H&E) for microscopic observation.

### 2.10. Statistical Analysis

Differences between groups were evaluated using the ordinary one-way ANOVA analysis, and statistical significance was considered when *p* < 0.05.

## 3. Results and Discussion

### 3.1. Polarizing Microscope

**Figure 2:**
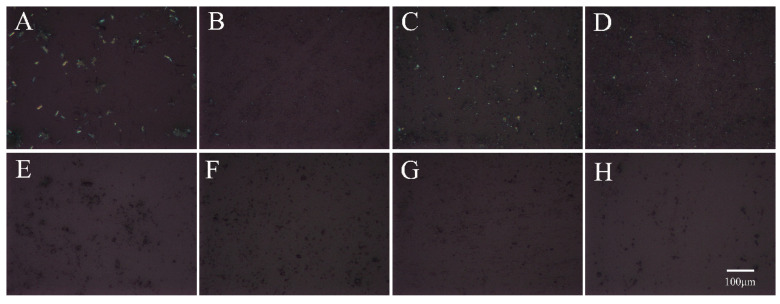
The PLM images of (**A**) ACM, (**B**) PM-Lys, (**C**) PM-Arg, (**D**) PM-His, (**E**) ACM-AM, (**F**) ACM-Lys, (**G**) ACM-Arg, and (**H**) ACM-His. The scale bar represents 100 μm.

As shown in Figure 2, the three PMs displayed a birefringence phenomenon, indicating that the substances existed in the crystalline state. In contrast, ACM-AM and three CAM systems did not show birefringence, suggesting the formation of an amorphous form.

### 3.2. PXRD

**Figure 3:**
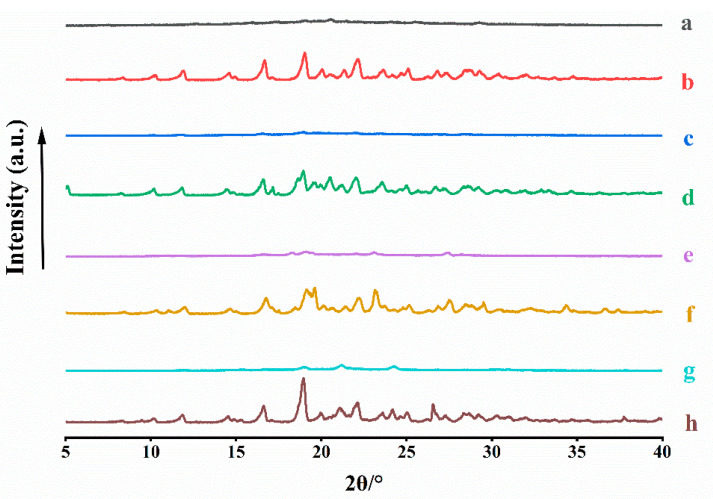
PXRD patterns of (a) ACM-AM, (b) ACM, (c) ACM-Lys, (d) PM-Lys, (e) ACM-Arg, (f) PM-Arg, (g) ACM-His, and (h) PM-His.

To confirm the formation of CAM systems, PXRD was performed. The patterns of ACM-AM, ACM, ACM-CAMs, and PMs are shown in Figure 3. ACM exhibited Bragg’s diffraction peaks at 2θ of 8.34°, 10.20°, 11.90°, 14.60°, 16.68°, 19.02°, 20.04°, 21.34°, 23.62°, and 25.08°, which was consistent with a previous report [22]. ACM-AM showed a broad halo. PMs displayed characteristic peaks of both ACM and amino acids [23]. However, no characteristic peaks could be observed in the patterns of ACM-CAMs, suggesting the successful preparation.

### 3.3. Temperature Differential Scanning Calorimetry

**Figure 4:**
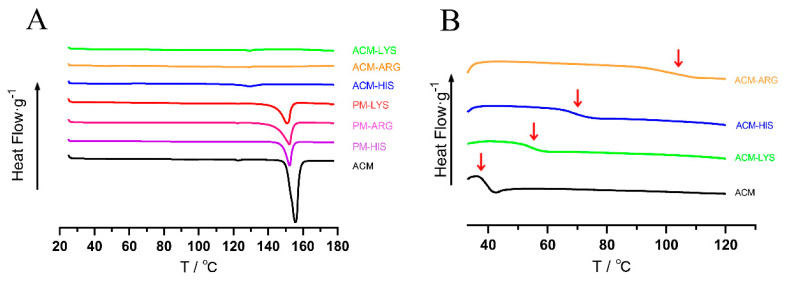
DSC thermograms (**A**) and the second heating DSC curves (**B**) of ACM-His, ACM-Lys, ACM-Arg, PM-His, PM-Lys, PM-Arg, and ACM.

As shown in Figure 4A, ACM had a significant melting peak at 153 °C. The melting peak of ACM in PMs was moved to 150 °C, due to the interaction with amino acids. No peaks were observed in ACM-Lys, ACM-Arg, or ACM-His, indicating the successful preparation of CAM systems.

*T_g_* is one of the characteristic parameters of amorphous systems. As shown in Figure 4B, all the systems only have one *T_g_*, indicating the formation of a single homogeneous amorphous phase. Concretely, ACM-AM had a *T_g_* of 37 °C, and the *T_g_* of ACM-Arg, ACM-His, and ACM-Lys increased to 105 °C, 70 °C, and 55 °C, respectively. The increased *T_g_* indicated the intermolecular interaction. In addition, the theoretical *T_g__(mix)_* of ACM-Lys, ACM-Arg, and ACM-His were then calculated using the Gordon–Taylor equation, and the *T_g__(mix)_* were 40.69 °C, 42.79 °C, and 37.00 °C, respectively. Because of the intermolecular interactions between ACM and amino acids, they were much lower than the actual *T_g_* values of the ACM CAM systems. According to previous reports, a high difference between *T_g__(mix)_* and actual *T_g_* tends to imply intermolecular interactions [24]. Therefore, we can conclude that ACM-Arg has the strongest intermolecular forces among the ACM-CAMs, followed by ACM-His and ACM-Lys.

### 3.4. Investigation of Molecular Interaction

#### 3.4.1. FTIR

**Figure 5:**
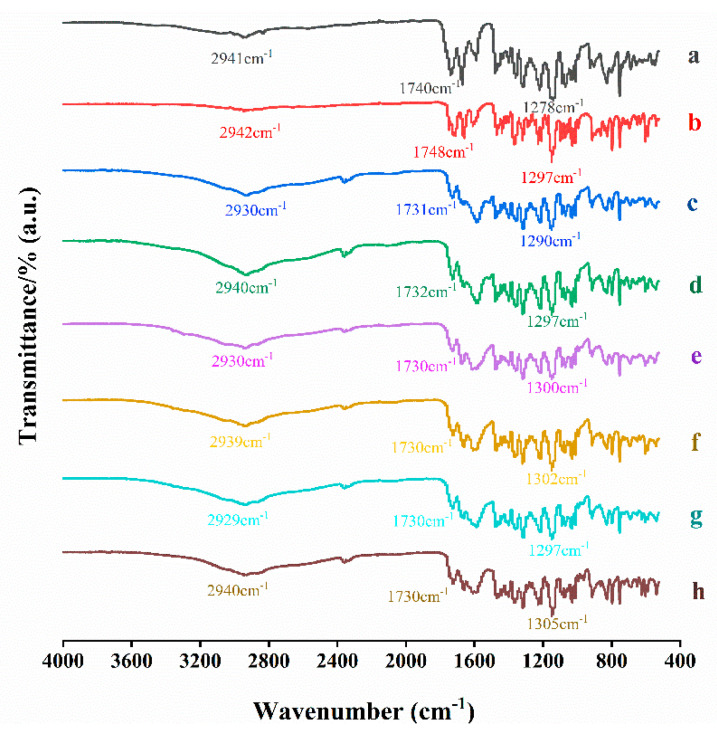
FTIR spectra of (a) ACM-AM, (b) ACM, (c) ACM-Lys, (d) PM-Lys, (e) ACM-Arg, (f) PM-Arg, (g) ACM-His, and (h) PM-His.

FTIR was performed to test whether molecular interactions exist between ACM and basic amino acids. As shown in Figure 5, the FTIR spectrum for ACM showed its characteristic peaks at 2942 cm^−1^, 1748 cm^−1^, and 1297 cm^−1^, which correspond to the stretching vibration of O−H, C=O, and C−O in carboxylic, respectively. In the spectrum for ACM-AM, the characteristic peaks were shifted to 2942 cm^−1^, 1748 cm^−1^, and 1278 cm^−1^, which may be due to the disruption of the crystal structure and reconfiguration of ACM molecules [25]. Compared with PMs, the stretching vibration peaks of O−H and C−O in ACM CAM systems showed a slight red shift, indicating that hydrogen bonding interactions exist between ACM and amino acids.

#### 3.4.2. Molecular Docking

**Figure 6:**
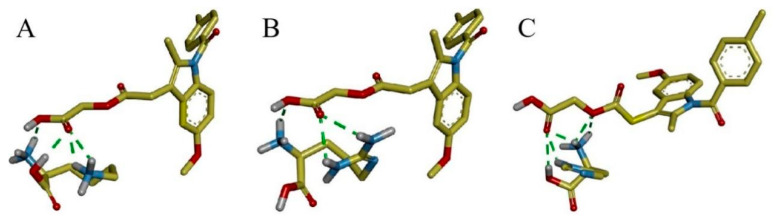
The molecular docking of the interaction between ACM and (**A**) Lys, (**B**) Arg, and (**C**) His. Red represents oxygen atoms, blue for nitrogen atoms, gray for hydrogen atoms, and the green dotted lines indicate hydrogen bonds formed between atoms.

Molecular docking was carried out to further investigate the molecular interactions between ACM and basic amino acids. The results are shown in Figure 6. The carboxyl group of ACM forms hydrogen bonds with the amino groups in Lys and Arg, as well as with the amino and hydroxyl groups in His. The intermolecular forces are consistent with FTIR. The magnitude of the binding energy reflects the strength of the intermolecular forces, and the binding energies for ACM-Lys, ACM-Arg, and ACM-His are −2.08 kcal/mol, −2.46 kcal/mol, and −2.13 kcal/mol, respectively. The prediction of the strength of the intermolecular forces is consistent with the *T_g_* results described in Section 3.3.

### 3.5. Dissolution Testing

**Figure 7:**
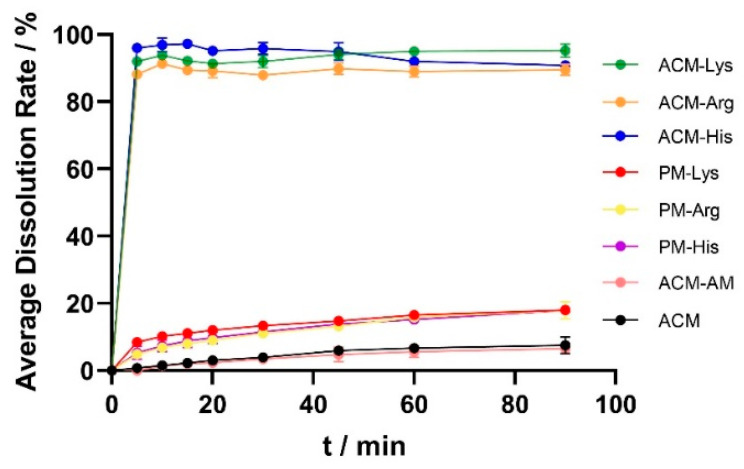
Dissolution profiles of ACM, ACM-AM, PMs, and ACM CAM systems.

The dissolution curves of ACM, ACM-AM, PMs, and ACM CAM systems are shown in Figure 7. The cumulative release of ACM was 7.53% at 60 min, and the dissolution did not increase after the formulation of ACM-AM (6.57% at 60 min), indicating that the neat amorphous counterpart could not improve the dissolution of ACM. In addition, the dissolution of PMs was slightly improved compared with that of ACM, which released 18% of the total drug. However, the ACM CAM systems showed complete dissolution within 5 min. The results showed that ACM CAM systems could improve the dissolution of ACM, probably due to the strong interaction between ACM and amino acids [24].

### 3.6. IDR

**Figure 8:**
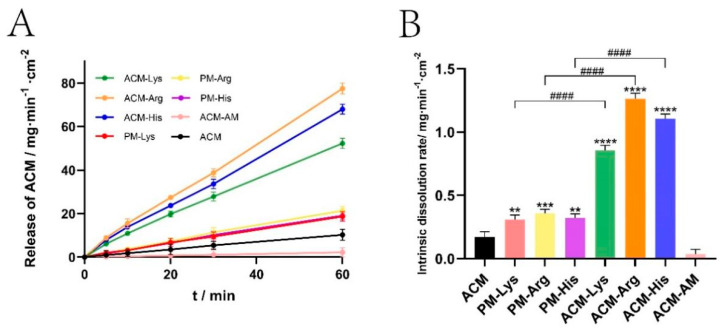
IDR curves (**A**) and the normalized dissolution rates (**B**) of ACM, ACM-AM, PMs, and ACM CAM systems (*n* = 3, ** *p* < 0.01, *** *p* < 0.001, **** *p* < 0.0001 vs. ACM and #### *p* < 0.0001 vs. PM groups).

The IDR can predict oral bioavailability to a certain extent [26]. Thus, for poorly water-soluble drugs, it is significant to conduct IDR tests. The IDR curves and the normalized dissolution rates are shown in Figure 8. The IDRs of ACM and ACM-AM were 0.1712 mg·min^−1^·cm^−2^ and 0.0373 mg·min^−1^·cm^−2^, respectively. For the PMs (PM-Lys, PM-Arg, and ACM-His), the IDRs were increased to 0.3087 mg·min^−1^·cm^−2^, 0.3565 mg·min^−1^·cm^−2^, and 0.3200 mg·min^−1^·cm^−2^, respectively. In addition, the IDRs of ACM CAM systems (ACM-Lys, ACM-Arg, and ACM-His) were further increased, which were 3.43, 7.39, and 6.46 times higher than those of ACM, respectively. The results indicated that basic amino acids as co-formers could improve the IDR of ACM.

From the results, it can be seen that ACM-Arg shows the highest IDR, followed by ACM-His and ACM-Lys. This may be attributed to the different strength of the intermolecular interaction between amino acids and ACM. According to a previous study [27], strong intermolecular interactions can prevent the recrystallization of components during dissolution. Therefore, Arg has the highest IDR because it has the strongest intermolecular forces.

### 3.7. Gastro-Protective Effects

#### 3.7.1. UIR

**Figure 9:**
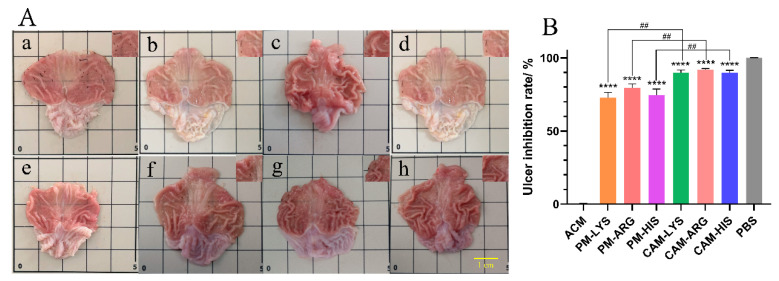
Stomach anatomy images (**A**) and the inhibition rate (**B**) of (**a**) ACM, (**b**) PM-Lys, (**c**) PM-Arg, (**d**) PM-His, (**e**) PBS, (**f**) CAM-Lys, (**g**) CAM-Arg, and (**h**) CAM-His. The scale bar represents 1 cm (*n* = 4, **** *p* < 0.0001 vs. ACM and ## *p* < 0.001 vs. PM groups).

To test the potential of ACM CAM systems in the protection of the stomach from gastric ulcers, the UIR was evaluated. The representative images of the stomachs and the UIR of each group are shown in Figure 9. The gastric mucosa of the blank control group was thick with more folds, light pink in color, and no ulcers or bleeding spots were observed. In contrast, the model group showed a looser and thinner gastric mucosa with fewer folds and a large number of bleeding spots spread over the whole gastric tissue. Moreover, the total area of ulcerative lesions in the model group was 10.171 mm^−2^, indicating that the gastric ulcer model was successfully established. PM-treated groups showed reduced gastric ulcers and visible bleeding spots. The areas of ulcerative lesions for PM-Lys-, PM-Arg-, and PM-His-treated groups reduced to 3.166 mm^−2^, 2.361 mm^−2^, and 2.841 mm^−2^, and the ulcer inhibition rate of those three groups was 72.7%, 79.3%, and 74.7%, respectively. As for ACM CAM-treated groups, ulcers and bleeding spots could not be observed. These groups (ACM-Lys, ACM-Arg, and ACM-His) had the smallest gastric ulcer areas of 1.108 mm^−2^, 0.983 mm^−2^, and 1.141 mm^−2^, with ulcer inhibition rates of 89.7%, 91.7%, and 89.9%, respectively.

The results above demonstrated that ACM CAM systems have significant ulcer mitigating effects (*p* < 0.05). According to a previous study [28], gastric ulcers caused by NSAIDs are related to their retention time in the gastric mucosa. In this study, the dissolution rate of ACM CAM systems was highly superior to that of PMs in the IDR test, which results in a shortened retention time in the mucosa. Thus, the improved ulcer mitigating effects of the ACM CAM systems might be due to the increased dissolution rate.

#### 3.7.2. The Histopathological Observation of the Stomach

**Figure 10:**
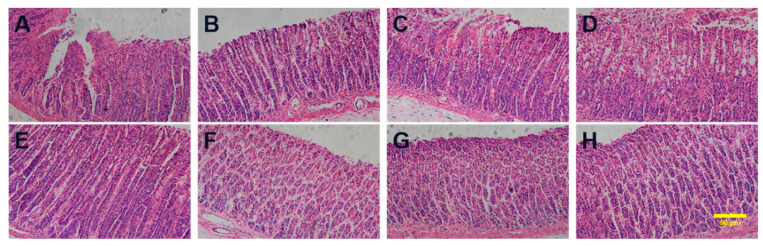
Histopathological examinations of (**A**) ACM, (**B**) PM-Lys, (**C**) PM-Arg, (**D**) PM-His, (**E**) PBS, (**F**) CAM-Lys, (**G**) CAM-Arg, and (**H**) CAM-His. The scale bar represents 50 μm.

Gastric mucosal damage is a typical symptom of gastric ulcers. The histopathological observation of the gastric tissues is shown in Figure 10. The normal gastric tissue had an intact gastric mucosa but did not show edema in the submucosa or epithelial cell detachment, congestion, or inflammatory cell infiltration. In the ACM-treated group, the gastric mucosa was damaged, with edema in the submucosa, mesenchymal congestion, and inflammatory cells infiltrated. In PM-treated groups, the intact gastric mucosa structure showed less congestion and inflammatory infiltration compared to the ACM-treated group. As for ACM CAM-treated groups, the gastric tissue was intact, no damage was observed, and less congestion or inflammatory infiltration was shown, which is similar to that of the blank control group. The results showed that the ACM CAM-treated group could almost eliminate the damage of ACM on the gastric tissue.

## 4. Conclusions

In this study, the feasibility of three basic amino acids (Lys, Arg, and His) as co-formers forming CAM systems with ACM was investigated. The results showed that ACM CAM systems were formed through hydrogen bonds between ACM and amino acids, which were confirmed by the analysis of *T_g_*, FTIR, and molecular docking. The ACM CAM systems showed an increased dissolution and IDR significantly. Furthermore, ACM CAM systems exhibited a great mitigation effect on the ACM-induced gastric ulcer with a UIR of up to almost 90%, probably due to the shortened retention time of ACM CAM systems on the gastric mucosa. Above all, this study provides a promising strategy for mitigating drug-induced side effects.

## Data Availability

The data presented in this study are available on request from the corresponding author.
